# Real-Time PCR and Quantitative Culture for *Mycoplasma pneumoniae* Load in Pharyngeal Swabs from Children at Preliminary Diagnosis and Discharge

**DOI:** 10.1155/2020/9814916

**Published:** 2020-01-03

**Authors:** Fei Zhao, Xuemei Guan, Jing Li, Liyong Liu, Jie Gong, Lihua He, Fanliang Meng, Jianzhong Zhang

**Affiliations:** ^1^National Institute for Communicable Disease Control and Prevention, Chinese Center for Disease Control and Prevention, State Key Laboratory of Infectious Disease Prevention and Control, Collaborative Innovation Center for Diagnosis and Treatment of Infectious Diseases, Beijing 102206, China; ^2^Affiliated Hospital of Beihua University, Jilin 132011, China; ^3^Office of Laboratory Management, Chinese Center for Disease Control and Prevention, Beijing 102206, China

## Abstract

**Background:**

Extensive studies have focused on the diagnosis and treatment of *Mycoplasma pneumoniae* infection; however, rare studies investigated the posttreatment conditions. We analyzed the carrying status of *M. pneumoniae* in the respiratory tract of children before and after treatment.

**Methods:**

Ninety-two children with *M. pneumoniae* pneumonia were included in this study. Clinical data were obtained from each patient, and pharyngeal swab sampling was performed at preliminary diagnosis and discharge. Real-time PCR and dilution quantitative culture were utilized to determine the DNA quantification and number of viable *M. pneumoniae* from samples collected upon preliminary diagnosis and discharge.

**Results:**

All the 92 cases showed DNA positivity upon preliminary diagnosis, serum IgM antibody was detected in 80 patients, and positivity of *M. pneumoniae* culture was observed in 82 cases. Upon discharge, the *M. pneumoniae* nucleotide and culture positivity were detected in 87 and 49 cases, respectively. The content of viable *M. pneumoniae* was 10–10^4^ CCU/mL and 10–10^2^ CCU/mL in the preliminary diagnosis samples and discharge samples, respectively.

**Conclusions:**

Real-time PCR was rapid and effective for the qualitative diagnosis of *M. pneumoniae* at the early stage, but it cannot be used to evaluate the prognosis of patients with *M. pneumoniae* infection. Quantitative analysis for *M. pneumoniae* DNA could not directly reflex the viable strain content.

## 1. Introduction

*Mycoplasma pneumoniae* is a major cause of respiratory tract infections in human, especially children and adolescents [1, 2]. It is responsible for about 10%–40% of the community acquired pneumonia (CAP) in children annually [3, 4]. In clinical practice, the agents used for treating *M. pneumoniae* infection mainly consist of macrolides, fluoroquinolones, and tetracycline antibiotics [5, 6]. Nowadays, macrolide antibiotics are preferred for treating *M. pneumoniae* infection in children with low toxicities, less adverse events, and high blood drug concentration [7, 8]. Although *M. pneumoniae* pneumonia (MPP) is usually considered a self-limited disease, it may trigger pulmonary complications that may progress into refractory *M. pneumoniae* pneumonia (RMPP), severe *M. pneumoniae* pneumonia (SMPP), and even lethal pneumonia [9, 10]. Meanwhile, some may present concurrent infection and multiple-organ infection, which results in poor prognosis [11, 12]. Therefore, *M. pneumoniae* infection is a heavy burden to the pediatric practice worldwide.

The diagnosis of *M. pneumoniae* infection in childhood is mainly relied on serology. In acute stage, the most frequently used method is based on determination of serum IgM antibody [13]. However, false-negativity may be generated in early stage in some patients due to poor IgM antibody titer. Then the second IgM antibody determination is necessary in a short term for the early diagnosis of cases suspected with RMPP [14]. Recently, nucleic acid amplification techniques (NAATs) represented by real-time PCR, with high sensitivity and specificity, have been gradually utilized in *M. pneumoniae* determination. Particularly, NAATs are crucial in early stage, in which the antibody titer is not adequate [15, 16].

The dissemination of viable *M. pneumoniae* is merely depending on aerosols, and infection models have been established in animals using aerosol inoculation [17]. Pathogens have been identified in the samples obtained from nose, throat, trachea, and sputum, which can be disseminated to a large scale through cough. Up to now, few studies have focused on the relationship between *M. pneumoniae* infection and dissemination. Meanwhile, rare studies have reported the prognosis of patients with *M. pneumoniae* infection after treatment [18]. Nilsson et al. [19] reported that *M. pneumoniae* DNA was still detected in the throat secretion in half of the patients about 7 weeks after disease onset. Moreover, certain cases showed persistent DNA positivity within several months. However, this scientific significance was hampered by the limitations of their study as the method could not tell the DNA of viable *M. pneumoniae* apart from nonviable ones [20]. Thus, methods that can accurately measure the amount of *M. pneumoniae* in throat are required to evaluate the clinical efficiency of treatment options. In this study, pharyngeal swab sampling was performed to the 92 children with *M. pneumoniae* pneumonia (MPP) before and after treatment, followed by real-time PCR, genotyping, and *M. pneumoniae* culture detection. We aim to investigate the carrier state of *M. pneumoniae* in the respiratory tract before and after treatment.

## 2. Material and Methods

### 2.1. Ethical Statement

Informed consent was obtained from at least one guardian of patients before enrolling this study. As the samples were collected in a noninvasive manner using a pharyngeal swab, the informed consent was obtained in an oral form from the guardian of each child. All the individuals were informed that the samples were only used for this clinical study. The study was approved by the Ethics Committee of the National Institute for Communicable Disease Control and Prevention, Chinese Center for Disease Control and Prevention (Beijing, China), and the Ethics Committee of the Affiliated Hospital of Beihua University (Jilin, China).

### 2.2. Patients

Ninety-two children with MPP admitted to Department of Pediatrics of Affiliated Hospital of Beihua University between January 2017 and March 2017 were included in this study. Patients with immune deficiency and/or received administration of antibiotics 2 weeks before diagnosis were excluded from this study. *M. pneumoniae* infection was diagnosed based on the demonstration of an IgM-specific anti-*M. pneumoniae* antibody titer of ≥1 : 160. All the patients received alternating treatment using intravenous injection of azithromycin (10 mg/kg, q.d., 5 days) and erythromycin (10 mg/kg, b.i.d., 3 days). All the patients were discharged from hospital upon remission of the symptoms, including elimination of positive signs and absorption of pulmonary lesions by chest film. After discharge, each patient was suggested to receive oral administration of azithromycin for 2-3 weeks.

### 2.3. Sample Collection

Pharyngeal swabs were collected from each patient upon preliminary diagnosis and labeled as A01 to A92, and upon discharge as B01 to B92, respectively. The samples were stored in *Mycoplasma* broth supplemented with 20% sterilized glycerol at −80°C upon collection and then transmitted to the National Institute for Communicable Disease Control and Prevention for subsequent analysis through cold chain ways.

### 2.4. Real-Time PCR

DNA was extracted from 200 *μ*l samples using QIAamp DNA MINI kit (QIAGEN, No. 51306) according to the manufacturer's instructions. Quantitative real-time PCR was performed according to our previous description [21]. The amplification conditions were as follows: 95°C for 3 min, followed by 45 cycles of 95°C for 15 sec and 59°C for 30 sec. The standard curve was established using the standard concentration nucleotides (2 copies/*μ*l to 2 × 10^6^ copies/*μ*l) of *M. pneumoniae* ATCC 29342 strain.

### 2.5. *M. pneumoniae* Culture and Identification of Clinical Samples

Clinical samples (200 *μ*l) were inoculated on *Mycoplasma* selective broth medium (OXOID, Thermo Fisher, NY, USA) at 37°C. The ATCC 29342 strains served as the positive control, and the blank culture served as the negative control. The remaining samples were stored using an Eppendorf tube (200 *μ*l) at −80°C until subsequent analysis. A yellow color on the medium with no turbidity was speculated to be *M. pneumoniae* positive. Bacterial contamination was considered in the presence of turbidity in the medium. The stored samples were subject to filtration using a 450 nm filter and then were cultured. Negativity was defined as no color changes within 5 weeks after culture. The color of medium was observed and the time interval between inoculation and *M. pneumoniae* positivity (TIIP) was recorded per day. Then 100 *μ*l positive samples were inoculated on the selective agar medium. After culturing at 37°C for 1 week, the colonies were observed under a stereomicroscope. The typical colonies were passaged, followed by DNA extraction using QIAamp DNA MINI kit (Qiagen, No. 51306). Then passaged *M. pneumoniae* identification was conducted using real-time PCR as previously described [22].

### 2.6. *M. pneumoniae* Quantitative Culture of Clinical Samples

Quantitative culture analysis of *M. pneumoniae* was performed to the cultivate positive samples collected both upon preliminary diagnosis and discharge using the liquid broth medium dilution method by color-changing units (CCUs). The samples were subject to dilution culture (10×) in liquid medium (6 gradients for each sample; triplicate for each gradient) and cultured at 37°C. The ATCC 29342 strains served as the positive control, while the blank culture served as negative control. The number of viable *M. pneumoniae* was performed by determining the CCU value. Two pairs of samples presenting bacterial contamination (A26/B26 and A28/B28) were dropped out due to bias in the quantitative analysis generated in the presence of filtration of sample using a 450 nm filter.

### 2.7. Typing of Multiple-Locus Variable-Number Tandem-Repeat Analysis (MLVA)

*M. pneumoniae* positive samples upon preliminary diagnosis and discharge were selected for the MLVA typing. MLVA typing was performed according to the previous description with moderate modifications method [23, 24]. The DNA of each *M. pneumoniae* isolate was utilized as the template for Multiplex PCR amplification-linked capillary electrophoresis of four loci (i.e., Mpn13-16) selected for multilocus variable-number tandem-repeat (VNTR) analysis.

### 2.8. Statistical Analysis

All the data were entered into Excel 2007 sheet, and SPSS 17.0 software was utilized for the data analysis. Chi square test was performed for the nucleotide and culture positivity of *M. pneumoniae* upon preliminary diagnosis and discharge, as well as the bacterial contamination rate at these two time points. Paired Student's *t*-test was used for the comparison of DNA load and viable *M. pneumoniae* content upon preliminary diagnosis and discharge. *P* value of less than 0.05 was considered to be statistically significant.

## 3. Results

### 3.1. Clinical Characteristics of Patients

Ninety-two children (male: 65; female: 27; age: 5–15 years) were included in this study. The mean hospitalization duration was 10.7 days. The examination findings upon preliminary diagnosis were as follows: temperature, 38.33 ± 1.21°C; and white blood cell, 8.81 ± 2.40 cells/L. Among these patients, 83 (90.2%) showed cough, and 21 (22.8%) showed sputum. All the 92 cases (100%) showed shadows after X-ray. Serum *M. pneumoniae* IgM antibody was detected in 80 patients (87.0%), among which 12 were IgM-negative during the preliminary diagnosis. About 4–6 days after preliminary diagnosis, serum IgM antibody was performed to the 12 cases, and all of them were IgM-positive.

### 3.2. Real-Time PCR for the *M. pneumoniae*

**Figure 1:**
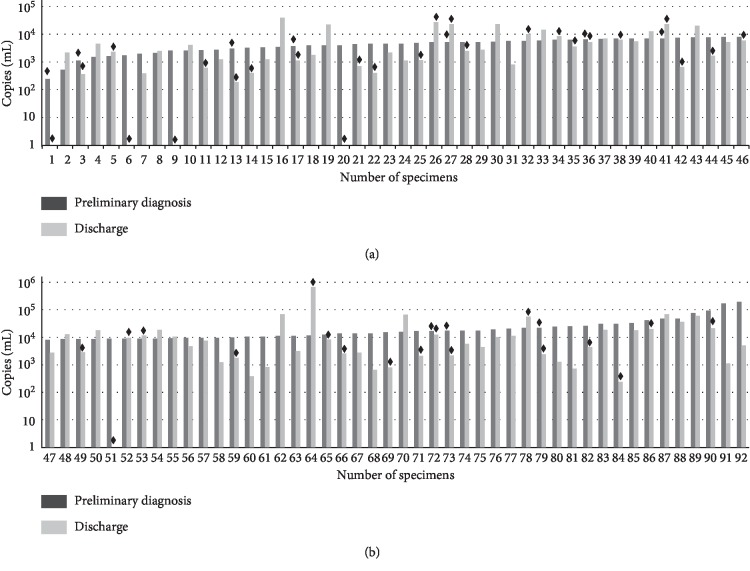
Comparison of DNA load in the *M. pneumoniae* obtained from the pharyngeal swab in 92 cases upon preliminary diagnosis and discharge. ◆: *M. pneumoniae* negative culture samples. The numbers of samples were arranged in an ascending order based on the DNA load of preliminary diagnosis.

No statistical differences were noticed in the detected DNA positivity of *M. pneumoniae* between that obtained upon preliminary diagnosis and that obtained upon discharge (100% vs. 94.6%, *χ*^2^ = 3.20, *P* > 0.05). *M. pneumoniae* DNA quantitative analysis indicated that the positive samples at the preliminary diagnosis showed no statistical differences compared with those obtained upon discharge (2.48 × 10^2^–1.95 × 10^5^ copies/mL vs. 1.86 × 10^2^–6.70 × 10^5^ copies/mL, *t* = 0.156, *P* > 0.05, Figure 1). The *M. pneumoniae* DNA quantity of 48 samples, including 5 negative samples, at preliminary diagnosis was higher than that at discharge. Twenty-eight samples showed comparable *M. pneumoniae* DNA quantity upon these two time points. The rest 16 samples showed lower *M. pneumoniae* DNA quantity at diagnosis compared to that of discharge. In each individual, elevation was defined as 2-fold increase in the DNA quantity.

### 3.3. *M. pneumoniae* Culture

At preliminary diagnosis, the positivity for *M. pneumoniae* culture was significantly higher than that of discharge (89.1% vs. 53.2%, *χ*^2^ = 31.03, *P* < 0.05). For the preliminary diagnosis, the concordance rate of culture and nucleic acid detection was 89.1% among the 92 cases, while that at the discharge was 58.7%. Nine pharyngeal swabs (i.e., A14, A17, A20, A21, A26, A28, A41, A44, and A45) showed bacterial contamination during *M. pneumoniae* culture at the preliminary diagnosis. Only two swabs (i.e., B14 and B17) showed contamination at discharge. There were statistical differences between the contamination rates in the samples obtained upon two time points (*χ*^2^ = 5.14, *P* < 0.05). The DNA quantitative analysis and *M. pneumoniae* culture results of all samples were shown in Table 1.

### 3.4. MLVA Typing

In total, 49 pairs of samples were *M. pneumoniae* positive both at preliminary diagnosis and at discharge. The MLVA typing of the strains was consistent at two time points in the 49 patients. Among these 98 *M. pneumoniae* isolates collected from 49 pairs of patients, the most common type was 4-5-7-2 MLVA type (87.8%, 86/98), followed by the MLVA 4-5-7-3 (8.2%, 8/98) and 4-4-7-2 (4.1%, 4/98), respectively. The MLVA genotypes of the strains were consistent in those isolated from the same patient upon preliminary diagnosis or discharge.

### 3.5. Quantitative Culture Analysis of Viable *M. pneumoniae* of Clinical Samples

**Figure 2:**
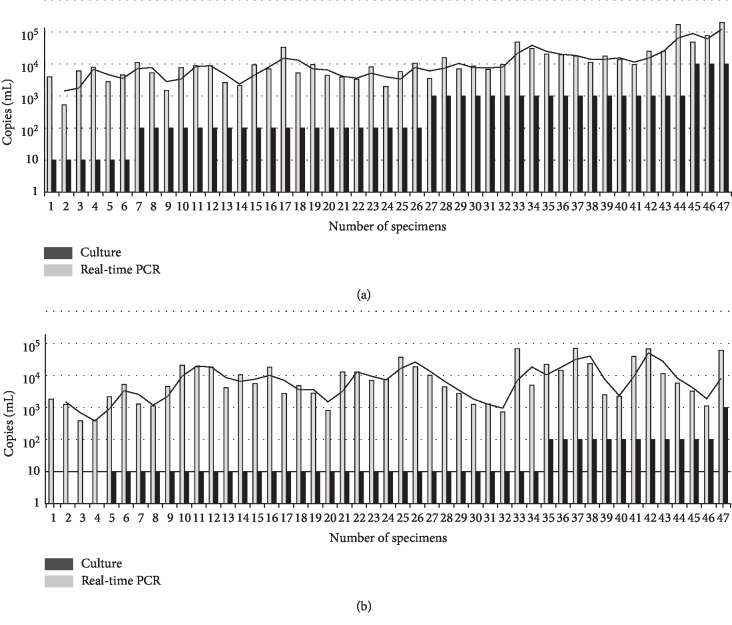
Comparison of viable *M. pneumoniae* load and DNA quantitative analysis in samples that were negative upon preliminary diagnosis (a) and discharge (b).

After excluding 2 pairs of samples with bacterial contamination (A26/B26 and A28/B28), CCU quantitative analysis was performed for the 47 pairs of *M. pneumoniae* culture positive samples. The content of viable *M. pneumoniae* in the preliminary diagnosis samples showed significant difference compared to that obtained upon discharge (10–10^4^ CCU/mL vs. 10–10^2^ CCU/mL, *t* = 2.980, *P* < 0.05). For the samples obtained from preliminary diagnosis, 6 samples showed a viable *M. pneumoniae* load of 10 CCU/mL, 20 showed a load of 10^2^ CCU/mL, 18 showed a load of 10^3^ CCU/mL, and 3 showed a load of 10^4^ CCU/mL. At discharge, 30 showed a load of 10 CCU/mL, 12 showed a load of 10^2^ CCU/mL, 1 showed a load of 10^3^ CCU/mL, and 4 showed negative results. Thirty-seven cases showed smaller *M. pneumoniae* CCU value at discharge compared to that of the preliminary diagnosis. Eight cases showed consistent CCU value at two time points, and only two showed higher CCU values at discharge (Table 2 and Figure 2).

### 3.6. TIIP

The shortest TIIP in the clinical samples was 7 days, and the latest TIIP was 30 days. Among the 92 cases, 80 showed a TIIP of 7–26 days for *M. pneumoniae* positivity at the preliminary diagnosis, while, at the discharge, 49 showed a TIIP of 16–30 days. Among the 47 pairs of *M. pneumoniae* positive samples, the TIIP of *M. pneumoniae* upon discharge was longer than that of the preliminary diagnosis in the samples of the same CCU. For the 8 patients with similar CCU value upon two time points (i.e., A03/B03, A06/B06, A32/B32, A37/B37, A40/B40, A70/B70, A73/B73, and A86/B86, Table 2), the TIIP of *M. pneumoniae* at discharge showed a 5.3-day delay compared to the preliminary diagnosis.

## 4. Discussion

In this study, 12 *M. pneumoniae* positive patients were IgM antibody negative, which then were IgM antibody positive about 4–6 days after preliminary diagnosis. This implied that the children were in an early stage of *M. pneumoniae* infection upon preliminary diagnosis, when serological test was not sufficient as there was no formation of IgM antibody or the titer of the antibody was not adequate. Compared with conventional serum IgM antibody determination, NAATs are superior in the early diagnosis of *M. pneumoniae* infection in order to improve the treatment efficiency and avoid antibiotics abuse.

Based on real-time PCR, Nilsson et al. [19] reported that about half of the subjects had detectable *M. pneumoniae* DNA in the oropharynx about 50 days, and even a longer duration of about 7 or more months. This indicated that *M. pneumoniae* DNA was available in oropharynx in a long term, which provided valuable information to the epidemiologic studies. The detection duration for DNA was more than 8 months in this report, which could not exclude the possibility of the *M. pneumoniae* reinfection. Moreover, DNA detection could not identify whether the target DNA fragment was from viable or nonviable organisms. Therefore, it is hard to discriminate the carrying status of the viable bacteria in each patient.

In this study, MLVA typing results were used to confirm infection of the same *M. pneumoniae* isolate. On this basis, possibilities of reinfection were excluded. The median hospitalization duration was 11 days, and the DNA positive rate at discharge was about 94.6%, which was similar with the previous study by Nilsson et al. [19] indicating up to 90% in the *M. pneumoniae* DNA positivity in throat about 10 days after infection. Thirty-three samples (40.2%) were positive for *M. pneumoniae* DNA at discharge; however, the culture results were negative. This implied a time delay in the clearance of DNA compared to the *M. pneumoniae* pathogen after treatment. These subjects were carriers of *M. pneumoniae* DNA other than viable isolates, who showed no infectious capability. In this study, freeze-thaw was performed once in the samples for the quantitative analysis. Among the *M. pneumoniae* positive samples, 4 cases showed *M. pneumoniae* negativity in the quantitative culture. This may be related to the potential effects of freeze-thaw on the viable bacteria number. Among the 47 patients that were *M. pneumoniae* positive at both preliminary diagnosis and discharge, 37 showed decrease in *M. pneumoniae* quantitative number at discharge (Table 2). Meanwhile, 43 patients were only *M. pneumoniae* positive at preliminary diagnosis (Figure 2). Taken together, 80 (88.9%) showed decrease of *M. pneumoniae* in number after treatment. Such trend could reflect the killing effects of antibiotics and immune clearance. The DNA used for the quantitative analysis was derived from viable *M. pneumoniae* and accumulated DNA from nonviable *M. pneumoniae*, which could not reflect the real carrying status of *M. pneumoniae* in each child at preliminary diagnosis or discharge. Therefore, the degradation and clearance of *M. pneumoniae* DNA are slow in *vivo*, and quantitative analysis is not adequate for the accurate determination.

The penicillin G and other bacteriostatic agents in the *Mycoplasma* selective medium can inhibit the majority of bacteria [25, 26]. Nevertheless, in few pharyngeal swabs, there were still bacteria resistant to penicillin, and there might be possibilities of bacterial contamination by the normal flora from the pharynx oralis. According to our experiences, the incidence of contamination by the bacteria in the swabs was in a range of 5% to 20%. In this study, significant differences were noticed in the bacterial contamination rate in the samples collected upon preliminary diagnosis and discharge (*χ*^2^ = 5.14, *P* < 0.05). This implied the clearance of penicillin-resistant bacteria in throat after macrolides antibiotics treatment. Indeed, it confirmed the side effects of antibiotics to the normal bacteria. On this basis, antibiotics were not recommended for the individuals with self-limited disease (e.g., mild *M. pneumoniae* infection) presenting mild symptoms [16, 27], in order to prevent the microbiota imbalance. For the analysis of CCU and TIIP of *M. pneumoniae*, obvious extension was observed in TIIP upon discharge compared with that of the preliminary diagnosis in the viable *M. pneumoniae* at the same CCU group (Table 3). Meanwhile, among the 8 cases with the same amount of *M. pneumoniae*, various delays were observed, and the extension of TIIP demonstrated growth inhibition of *M. pneumoniae*. Generally, in the absence of nonendogenous inhibiting agents, the effects of endogenous components on the culture of *M. pneumoniae* were nearly the same in a certain case. In this study, the endogenous components responsible for the delay of bacterial culture were likely to be the metabolites of macrolides antibiotics. This indicated that macrolides antibiotics and the metabolites showed inhibiting effects on *M. pneumoniae*.

In this study, we focused on the quantitative analysis of *M. pneumoniae* DNA and culture using same clinical samples. The clinical symptoms of the patients showed obvious remission after anti-infection therapy for *M. pneumoniae*, which was mainly characterized by *M. pneumoniae* clearance in about 40% of the patients after discharge, and decline of *M. pneumoniae* together with control of pathogenic bacteria in 60% of the patients. The clinical manifestations of patients with *M. pneumoniae* infection were featured by dry cough and small amount of sputum [27, 28]. Our data showed that 62 patients (67.4%) showed dry cough. For the patients in acute stage with typical symptoms and a large amount of *M. pneumoniae*, cough serves as an important source of infection. In spite of the fact that 60% of the patients were *M. pneumoniae* carriers of a small amount at discharge, these patients were symptom-free with very lower infectious capacity compared to that before treatment. Nevertheless, it is not sure whether these individuals are infectious or *M. pneumoniae* carriers, and then further studies are required.

There are limitations in our study. Firstly, it is not possible to follow up all the patients after discharge, and then the carrying status of the viable *M. pneumoniae* in the throat was not investigated in these patients. Secondly, we collected the samples using pharyngeal swabs, which may trigger bias in the amount of bacteria in the lower respiratory tract infection induced by *M. pneumoniae*. BALF samples may be ideal for this study. On this basis, further studies involving more accurate data are required.

In conclusion, we considered that DNA technique was rapid and effective for the qualitative diagnosis of *M. pneumoniae* at the early stage. However, it cannot be used to evaluate the prognosis of patients with *M. pneumoniae* infection, as well as the quantitative and qualitative analyses of *M. pneumoniae*. Qualitative culture was required to investigate the status of *M. pneumoniae*.

The numbers of specimens were arranged in an ascending order based on the number of viable bacteria.

## Figures and Tables

**Table 1:** Positive rates of samples with various *M. pneumoniae* DNA loads.

DNA load (copies/mL)	Positivity	
	Preliminary diagnosis	Discharge^*∗*^
<10^3^	50.0% (1/2)	40.0% (6/15)
10^3^–10^4^	89.5% (51/57)	55.6% (25/45)
>10^4^	91.0% (30/33)	66.7% (18/27)

^*∗*^Five DNA negative samples were excluded. The positivity was defined as the ratio of positive samples to total samples.

**Table 2:** *M. pneumoniae* DNA quantitative analysis, number of viable bacteria, and culture duration of the 47 pairs of positive samples.

Preliminary diagnosis				Discharge			
Patient no.	DNA load (copies/mL)	Viable count (CCU/mL)	TIIP (d)	Patient no.	DNA load (copies/mL)	Viable count (CCU/mL)	TIIP (d)
A002	5.96 × 10^3^	10	20	B002	1.45 × 10^4^	10^2^	23
A003	5.28 × 10^2^	10	19	B003	2.16 × 10^3^	10	24
A005	1.76 × 10^4^	10^3^	17	B005	4.36 × 10^3^	10	21
A006	2.79 × 10^3^	10	17	B006	1.26 × 10^3^	10	21
A008	6.94 × 10^3^	10^2^	19	B008	5.57 × 10^3^	10	25
A009	1.97 × 10^3^	10^2^	21	B009	3.82 × 02	N	N
A011	1.05 × 10^4^	10^2^	21	B011	3.83 × 02	N	N
A012	9.60 × 10^3^	10^3^	14	B012	1.23 × 10^3^	10	22
A016	2.45 × 10^4^	10^3^	8	B016	1.27 × 10^3^	10	24
A021	5.75 × 10^3^	10^2^	17	B021	8.12 × 02	10	29
A024	3.32 × 10^3^	10^2^	24	B024	1.24 × 10^3^	N	N
A029	2.62 × 10^3^	10^2^	19	B029	4.11 × 10^3^	10	25
A032	4.49 × 10^3^	10	22	B032	1.11 × 10^3^	10	28
A035	3.90 × 10^3^	10^2^	14	B035	1.82 × 10^3^	N	N
A037	5.30 × 10^3^	10^2^	12	B037	2.30 × 10^4^	10^2^	20
A040	4.45 × 10^3^	10^2^	18	B040	2.21 × 10^3^	10^2^	25
A041	9.56 × 10^3^	10^3^	18	B041	7.46 × 10^3^	10	25
A042	6.69 × 10^3^	10^3^	15	B042	6.98 × 10^3^	10	23
A043	3.51 × 10^3^	10^3^	18	B043	3.84 × 10^4^	10^2^	19
A045	7.67 × 10^3^	10^2^	14	B045	2.04 × 10^4^	10	21
A047	6.95 × 10^3^	10^3^	17	B047	1.26 × 10^4^	10	24
A051	8.74 × 10^3^	10^2^	18	B051	1.77 × 10^4^	10	22
A053	8.91 × 10^3^	10^2^	13	B053	1.86 × 10^4^	10	19
A055	1.75 × 10^4^	10^3^	12	B055	5.74 × 10^3^	10^2^	26
A056	1.56 × 10^4^	10^3^	14	B056	6.63 × 10^4^	10^2^	15
A058	4.75 × 10^4^	10^4^	18	B058	6.78 × 10^4^	10	20
A059	4.81 × 10^4^	10^3^	8	B059	3.65 × 10^4^	10	17
A060	9.52 × 10^3^	10^2^	13	B060	4.73 × 10^3^	10	27
A061	3.01 × 10^4^	10^3^	10	B061	1.84 × 10^4^	10	22
A062	5.22 × 10^3^	10^2^	16	B062	2.72 × 10^3^	10	25
A065	2.04 × 10^4^	10^3^	13	B065	1.14 × 10^4^	10^2^	23
A068	8.46 × 10^3^	10^3^	16	B068	1.28 × 10^4^	10	21
A069	1.50 × 10^3^	10^2^	21	B069	4.47 × 10^3^	10	23
A070	7.85 × 10^3^	10	16	B070	5.18 × 10^3^	10	25
A072	1.94 × 10^4^	10^3^	16	B072	9.99 × 10^3^	10	21
A073	1.11 × 10^4^	10^2^	19	B073	6.89 × 10^4^	10^2^	20
A074	1.36 × 10^4^	10^3^	14	B074	2.72 × 10^3^	10	22
A075	1.95 × 10^5^	10^4^	9	B075	4.95 × 10^3^	10	22
A076	9.32 × 10^3^	10^2^	13	B076	1.04 × 10^4^	10	25
A080	1.12 × 10^4^	10^3^	10	B080	3.18 × 10^3^	10^2^	22
A081	3.93 × 10^3^	10	26	B081	2.20 × 10^4^	10^2^	19
A086	2.13 × 10^3^	10^2^	25	B086	2.47 × 10^3^	10^2^	27
A087	2.49 × 10^4^	10^3^	8	B087	7.18 × 02	10	25
A088	8.03 × 10^3^	10^2^	18	B088	2.78 × 10^3^	10	20
A089	7.68 × 10^4^	10^4^	9	B089	6.05 × 10^4^	10^3^	16
A090	1.67 × 10^5^	10^3^	7	B090	1.11 × 10^3^	10^2^	26
A091	3.30 × 10^4^	10^2^	12	B091	1.80 × 10^4^	10	18

N: negative.

**Table 3:** Comparison of TIIP of *M. pneumoniae* culture quantitative analysis upon preliminary diagnosis and discharge.

Viable bacteria (CCU/mL)	Positive sample		TIIP		TIIP, median		TIIP, mean	
	Preliminary diagnosis	Discharge	Preliminary diagnosis	Discharge	Preliminary diagnosis	Discharge	Preliminary diagnosis	Discharge
10	6	30	16–26	17–29	19.5	22.5	20	22.9
10^2^	20	12	12–25	23–27	18	22	17.4	22.1
10^3^	18	1	7–18	16	14	16	13.1	16
10^4^	3	0	9–18	—	9	—	12	—

—: not calculated.

## Data Availability

All the data were available upon appropriate request.
